# Pre-natal manifestation of systemic developmental abnormalities in spinal muscular atrophy

**DOI:** 10.1093/hmg/ddaa146

**Published:** 2020-07-09

**Authors:** Anna A L Motyl, Kiterie M E Faller, Ewout J N Groen, Rachel A Kline, Samantha L Eaton, Leire M Ledahawsky, Helena Chaytow, Douglas J Lamont, Thomas M Wishart, Yu-Ting Huang, Thomas H Gillingwater

**Affiliations:** Edinburgh Medical School: Biomedical Sciences, University of Edinburgh, Edinburgh EH8 9XD, UK; Euan MacDonald Centre for Motor Neurone Disease Research, University of Edinburgh, Edinburgh EH16 4SB, UK; Royal (Dick) School of Veterinary Studies, University of Edinburgh, Edinburgh EH25 9RG, UK; UMC Utrecht Brain Center, University Medical Center, Utrecht 3584 CG, The Netherlands; Euan MacDonald Centre for Motor Neurone Disease Research, University of Edinburgh, Edinburgh EH16 4SB, UK; The Roslin Institute, University of Edinburgh, Easter Bush, Midlothian EH25 9RG, UK; Euan MacDonald Centre for Motor Neurone Disease Research, University of Edinburgh, Edinburgh EH16 4SB, UK; The Roslin Institute, University of Edinburgh, Easter Bush, Midlothian EH25 9RG, UK; Edinburgh Medical School: Biomedical Sciences, University of Edinburgh, Edinburgh EH8 9XD, UK; Euan MacDonald Centre for Motor Neurone Disease Research, University of Edinburgh, Edinburgh EH16 4SB, UK; Edinburgh Medical School: Biomedical Sciences, University of Edinburgh, Edinburgh EH8 9XD, UK; Euan MacDonald Centre for Motor Neurone Disease Research, University of Edinburgh, Edinburgh EH16 4SB, UK; FingerPrints Proteomics Facility, University of Dundee, DD1 5EH, UK; Euan MacDonald Centre for Motor Neurone Disease Research, University of Edinburgh, Edinburgh EH16 4SB, UK; The Roslin Institute, University of Edinburgh, Easter Bush, Midlothian EH25 9RG, UK; Edinburgh Medical School: Biomedical Sciences, University of Edinburgh, Edinburgh EH8 9XD, UK; Euan MacDonald Centre for Motor Neurone Disease Research, University of Edinburgh, Edinburgh EH16 4SB, UK; Edinburgh Medical School: Biomedical Sciences, University of Edinburgh, Edinburgh EH8 9XD, UK; Euan MacDonald Centre for Motor Neurone Disease Research, University of Edinburgh, Edinburgh EH16 4SB, UK

## Abstract

Spinal muscular atrophy (SMA) is a neuromuscular disease caused by mutations in *survival motor neuron 1* (*SMN1*). SMN-restoring therapies have recently emerged; however, preclinical and clinical studies revealed a limited therapeutic time window and systemic aspects of the disease. This raises a fundamental question of whether SMA has presymptomatic, developmental components to disease pathogenesis. We have addressed this by combining micro-computed tomography (μCT) and comparative proteomics to examine systemic pre-symptomatic changes in a prenatal mouse model of SMA. Quantitative μCT analyses revealed that SMA embryos were significantly smaller than littermate controls, indicative of general developmental delay. More specifically, cardiac ventricles were smaller in SMA hearts, whilst liver and brain remained unaffected. In order to explore the molecular consequences of SMN depletion during development, we generated comprehensive, high-resolution, proteomic profiles of neuronal and non-neuronal organs in SMA mouse embryos. Significant molecular perturbations were observed in all organs examined, highlighting tissue-specific prenatal molecular phenotypes in SMA. Together, our data demonstrate considerable systemic changes at an early, presymptomatic stage in SMA mice, revealing a significant developmental component to SMA pathogenesis.

## Introduction

Spinal muscular atrophy (SMA) is the second most common autosomal recessive disorder in humans, after cystic fibrosis: with 2% of people carrying an SMA-associated mutation, this disease affects 1 in 6000–10 000 live births and is the leading genetic cause of infant death (1,2). SMA is primarily a neurodegenerative disease characterized by loss of lower motor neurons and muscle atrophy (3–5). In 98% of cases, SMA arises from a homozygous mutation in the *survival motor neuron 1* gene (*SMN1*) (6), leading to insufficient production of full-length SMN protein. SMN is ubiquitously expressed in all cell types, including during development and is crucial for survival (7,8). Mouse embryos in which *Smn* has been genetically knocked out die around the peri-implantation stage (9).

In late 2016, Spinraza® (nusinersen) became the first FDA-approved drug to treat SMA (5,10–14). The FDA subsequently approved Zolgensma® (onasemnogene abeparvovec-xioi) in May 2019 (15,16). Both of these treatments work by increasing the levels of full-length SMN protein, the former through an antisense oligonucleotide and the latter using AAV9-delivered gene replacement therapy. However, despite the success of both Spinraza® and Zolgensma® in improving outcomes in SMA patients, their efficacy is highly variable from one patient to another, and they modify disease course rather than providing a cure (10,15,16).

One likely explanation for the modest efficacy of Spinraza® and Zolgensma® in some patients is the timing and targeting of therapy delivery. Thus, results from clinical trials suggest that treatment should be performed as early as possible to obtain maximal therapeutic benefit (10,17). This finding is supported by pre-clinical animal studies, which have revealed a narrow postnatal time window for the successful restoration of SMN in SMA mice (18–20). Moreover, several lines of evidence suggest that systemic delivery of therapies, rather than targeting of motor neurons in the spinal cord, is required for maximal therapeutic benefit (21–23).

Despite our growing awareness of the need for early therapeutic intervention in SMA, we still do not know where and when the disease first manifests. The majority of pre-clinical studies have been performed in post-natal animals. However, several studies support the hypothesis that SMA, particularly in its most severe forms, may present with pre-symptomatic, prenatal developmental phenotypes (24–27). To address this important issue, we have undertaken a comprehensive morphological and proteomic characterization of systemic features of SMA at a pre-symptomatic, prenatal stage in the Taiwanese mouse model of severe SMA. We report the presence of morphological and molecular changes across multiple tissues and organs at E14.5, thereby revealing significant pre-symptomatic developmental phenotypes in SMA, and provide access to freely available morphological and molecular datasets of SMA embryos that can be utilized for further research.

## Results

Most studies on pre-symptomatic SMA in the Taiwanese mouse model have focused on postnatal phenotypes. Here, we investigated disease manifestation during embryonic development at E14.5, approximately 13 days prior to overt neuromuscular symptom development. To better understand the systemic nature of disease at this time point of development, we examined a range of organs, including the brain, spinal cord, liver, heart and skeletal muscle.

### SMA mouse embryos are smaller than littermate controls

**Figure 1:**
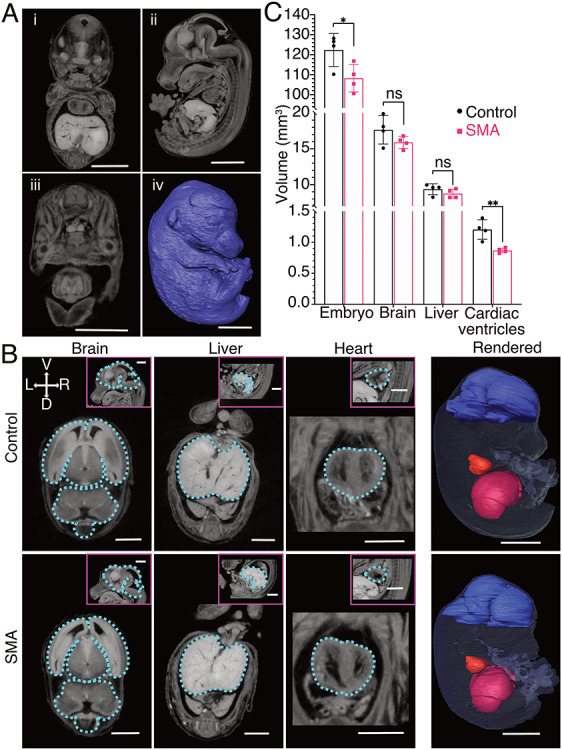
Anatomical differences in SMA mouse embryos at E14.5 revealed using μCT scans. (**A**) Representative 3D views of a control embryo from μCT scans: (**i**) coronal, (**ii**) sagittal, (**iii**) transverse, (**iv**) surface-rendered, scale bars = 2 mm. (**B**) Transverse images representative of the manual segmentation performed for each organ (outlined in blue), pink boxes show the corresponding sagittal view, scale bars = 1 mm. The far-right column shows surface rendering of the computed volumes for each region of interest, scale bars = 2 mm. (**C**) Volumetric data represented as a bar chart (mean, SD, *n* = 4 per genotype, ^*^P < 0.05, ^*^^*^P < 0.01, unpaired t-test).

We first asked whether anatomical defects could be detected in SMA embryos compared to littermate controls. To do so, we performed micro-computed tomography (μCT) imaging of whole embryos from a single litter of mice at E14.5 (Fig. 1A, i-iv). Initial anatomical, qualitative assessment of the embryos confirmed the presence and standard location of all major body organs and systems in both SMA and littermate animals. Using the scans, we then segmented the images to facilitate volumetric measurement and 3D reconstruction of regions of interest (Supplementary Material, Movie 1). We measured the volumes of whole embryos as well as brain, liver and the cardiac ventricles (Fig. 1B, Supplementary Material, Movies 2–5). Volumetric quantification revealed that SMA embryos were significantly smaller than their littermate controls (Fig. 1C; unpaired t-test, *n* = 4, *P* = 0.0396). Volume measurements of liver and brain revealed no difference between SMA and control embryos, whereas the cardiac ventricles were significantly smaller in the SMA animals (Fig. 1C; unpaired t-test, *n* = 4, *P* = 0.0059). Taken together, these experiments showed that SMA embryos differ from controls in total body size, suggesting a developmental delay in SMA embryos compared to littermate controls, and that internal organs are differentially affected.

### Cardiac ventricles do not show overt pathology

**Figure 2:**
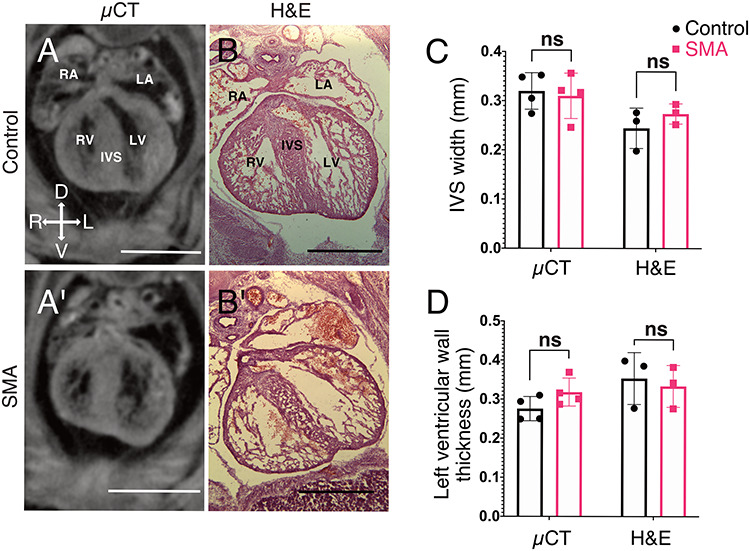
Absence of overt pathological defects in cardiac ventricles of SMA mouse embryos. (**A,A’**) Representative orientation-adjusted transverse μCT scans used for morphometric measurements. (**B,B’**) Representative HE-stained transverse cryosections used for morphometric measurements. IVS = interventricular septum, RV = right ventricle, LV = left ventricle, RA = right atrium, LA = left atrium, scale bars = 1 mm. (**C**) and (**D**) Morphometric data presented as bar charts showing no changes in morphological parameters previously used to identify degenerative cardiac pathology at later stages of SMA (SD, *n* = 4 per genotype for μCT data, *n* = 3 per genotype for cryosections, ^*^P < 0.05, unpaired t-test).

As cardiac ventricles were found to be significantly smaller in SMA mice (Fig. 1C), we next examined whether they exhibited any structural changes known to be representative of cardiac pathology, and which are known to occur in the heart of SMA mice at later developmental stages. For example, Shababi *et al.* (28) described thinning of the interventricular septum (IVS) in the E17.5 heart of SMA embryos of the same model. Using orientation-adjusted transverse μCT images, we performed morphometric measurements of the ventricles. We examined the width of the IVS and thickness of the left ventricular wall. Despite the smaller volume of SMA hearts at E14.5, no overt structural defects were identified (Fig. 2A, A’, C and D). To verify this finding, we performed the same measurements on H&E-stained transverse cryosections. Again, structural defects were not observed in the heart (Fig. 2B, B’, C and D), suggesting the presence of a developmental delay, rather than overt cardiac pathology, at this stage of embryonic development.

### Mass spectrometry reveals widespread proteomic changes

**Figure 3:**
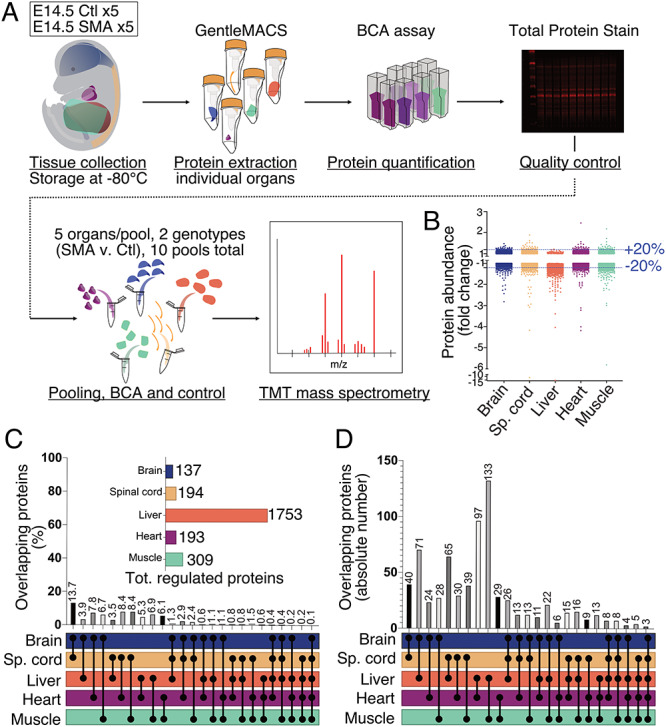
TMT mass spectrometry revealed widespread, organ-specific changes in the proteome of SMA mouse embryos at E14.5. (**A**) Schematic representation of sample processing and experimental pipeline for the proteomics screen, Ctl stands for Control embryos. (**B**) Dotplot of all protein IDs mapped in IPA for each organ of interest. Dotted blue lines show the 20% up- and down-regulated threshold. (**C**) Bar chart showing the percentage of overlapping protein IDs dysregulated by 20% or more for every possible combination of organs. The horizontal bar chart shows the size of each set of dysregulated proteins used to identify overlaps. (**D**) Bar chart showing the absolute number of overlapping protein IDs dysregulated by 20% or more for every combination of organs possible.

Having identified developmental differences between SMA and control embryos in mice at the morphological level, we next sought to establish whether developmental defects resulting from SMN depletion were occurring at a molecular level. Functionally, SMN is known to play key roles in regulating both transcription, where it participates in small nuclear ribonucleoprotein formation and is a component of the spliceosome (29), and translation (30). For this reason, we performed a proteomics screen to examine molecular differences at the level of the proteome, in preference to using RNA sequencing or microarray. For quantitative and comparative analyses, pooled samples of E14.5 brains, spinal cords, livers, hearts and skeletal abdominal muscles were processed by 10-plex tandem mass tagging (TMT) mass spectrometry (Fig. 3A).

Quality-checked (see Methods) identified proteins and abundance values were filtered to retain only proteins identified by two or more unique peptides. The ratios of SMA versus control abundance for each protein in every tissue were calculated and used as input for subsequent bioinformatic analysis using ingenuity pathway analysis (IPA). Out of a total of 7185 inputted ratios, the software returned 6701 mapped protein identifications. For additional stringency, all further analyses were performed only on these mapped IDs returned by IPA. A cut-off of 20%-fold change relative to controls (expressed as fold change of +1.2 or −1.2) was used to determine differentially-expressed proteins in SMA organs compared to controls. These analyses revealed widespread disruption to the proteome in SMA mice at E14.5, with all organs showing molecular effects of low levels of SMN at this stage of embryonic development (Fig. 3B, Supplementary Material, Table S1). Of all organs examined, the liver had the highest number of differentially-expressed proteins (1753) in SMA mice. Assessment of proteome differences across all organs revealed that the majority of proteins were downregulated, rather than upregulated, in SMA tissues (Supplementary Material, Table S1, Figure 3B).

### Changes in the proteome are organ specific

To examine the extent to which proteomic changes occurring downstream of low SMN levels were shared or divergent between different tissues and organs, we then determined the extent of overlap of proteome changes across combinations of two, three, four or all five organs studied (Fig. 3C and D). Brain and spinal cord shared the highest similarity of regulated proteins (13.7%), as might be expected given their similar cellular composition. However, in all of the other combinations of organs examined, the extent of overlap between proteomic changes was less than 10% (Fig. 3C). For example, a comparison of the SMN-modified proteome between spinal cord and heart showed only an 8.4% overlap. Likewise, comparison of the SMN-modified proteome between skeletal muscle and heart revealed a 6.1% overlap. In total, only 0.1% of proteomic changes downstream of SMN were conserved across all five organs and tissues examined (Fig. 3C). When considering the absolute numbers of overlapping proteins, only three individual proteins (GEMIN8, DDX20/GEMIN3 and HPS4) were significantly modified across all tissues and organs examined (Fig. 3D, Supplementary Material, Table S2). GEMIN8 and DDX20 are known SMN interactors, thereby providing internal controls to validate the proteomics data (31,32).

Although the pattern of developmental molecular disruption was heterogeneous between organs and tissues in SMA mouse embryos at the level of individual proteins, it remained possible that similar cellular pathways were being affected. Therefore, to gain a better understanding of the functional consequences of low levels of SMN, we used IPA to identify canonical pathways that were affected in each organ (see Materials and Methods and Supplementary Material, Table S3).

**Figure 4:**
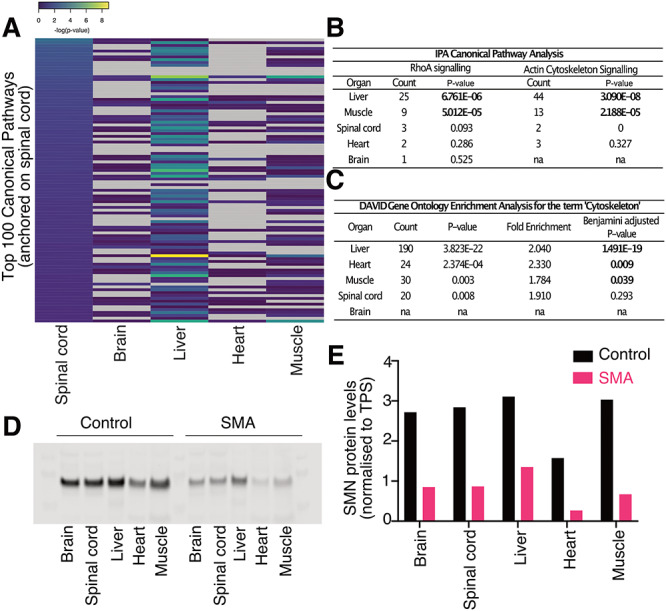
Organ/tissue-specific SMN expression and molecular pathway disruption. (**A**) Heatmap anchored on the top 100 affected canonical pathways of the spinal cord (according to -log(*P*-value) for each pathway identified), as generated by IPA. Pathways for which *P*-values could not be calculated in given organs are shown in grey. Note the lack of consistency of changes across each of the different tissues/organs. (**B**) Protein count and *P*-values (Fisher’s exact test, P^*^ < 0.05 shown in bold) reported by IPA for enrichment of the RhoA and Actin Cytoskeleton signalling pathways in each organ of interest. (**C**) Gene ontology enrichment analysis reported by DAVID for the term ‘cytoskeleton’ (Fisher’s exact test, significance reached for Benjamini-adjusted *P* < 0.05, shown in bold). (**D**) Western blot for SMN on pools of embryonic organs used for mass spectrometry (*n* = 5 per genotype). (**E**) Bar chart representing the quantification of the above western blot. SMN protein levels were normalized to total protein stain signal for each lane.

Important pathways for embryonic development were amongst the most affected ones in each organ, such as ephrin receptor signalling in the liver and PFKFB4 (6-phosphofructo-2-kinase/fructose-2,6-biphosphatase 4) signalling in the spinal cord, with a -log(*P*-value) of 10.2 and 3.16, respectively (Supplementary Material, Table S3). However, when we selected the top 100 most affected canonical pathways in the spinal cord and searched for similar pathway changes across the other organs and tissues, there was very little overlap. Thus, no identified canonical pathway was affected by the same magnitude across all five organs (Fig. 4A and Supplementary Material, Table S2). Heatmaps anchored to the top 100 affected canonical pathways of the other four organs further demonstrated the lack of commonality between affected pathways (Supplementary Material, Figure S1). Taken together, these detailed comparative proteomic analyses revealed a striking lack of overlap between the number or identity of dysregulated proteins and disrupted canonical pathways between distinct tissues and organs. Thus, these data demonstrate that at E14.5, SMN insufficiency causes a wide range of downstream molecular defects, which are highly organ specific. As a result, it may not always be possible to predict the molecular consequences of SMN depletion in one specific tissue or organ based on the profile observed in another.

Of particular relevance to SMA, RhoA signalling was identified in the canonical pathway analysis performed by IPA (33). Again, individual organs were affected differently: brain, spinal cord and heart only had one, three and two dysregulated proteins associated with the pathway, respectively. In contrast, the liver had 25 and the muscle had nine dysregulated proteins, corresponding to *P*-values of 6.761E-06 and 5.012E-05 for protein enrichment (Fig. 4B). Similarly, actin cytoskeleton signalling, also identified by IPA, was significantly disrupted in the liver (*P*-value of 3.090E-08 and 44 proteins) and skeletal muscle (*P*-value of 2.188E-05 for 13 proteins), but with only a few annotated proteins in the spinal cord and heart (Fig. 4B). To explore this further, we used The Database for Annotation, Visualization and Integrated Discovery (DAVID) to perform functional annotation of the differentially-expressed proteins from each organ. The term ‘cytoskeleton’ was prevalent in the gene ontology enrichment analysis of all organs apart from the brain, which reflects its overall low number of dysregulated proteins (Fig. 4C).

### SMN protein levels do not correspond to the number of dysregulated proteins

To determine the extent to which organ-specific responses in SMA may result from differing absolute levels of SMN protein between tissues and organs, we compared SMN levels across each of the organs in our study using quantitative western blot (34,35). SMN protein levels were measured in embryonic samples pooled from the original protein extracts used for mass spectrometry in order to allow direct correlative comparison of findings (Fig. 4D). The liver revealed the highest relative levels of SMN protein in both control and SMA samples at E14.5 (Fig. 4E). SMN levels present in control livers suggests that the liver requires high amounts of SMN protein at this embryonic age and may therefore explain, at least in part, the finding that it was the most affected organ in our proteomic screen. However, there was no relationship between the number of dysregulated proteins in each organ and SMN levels in control animals, nor was there a relationship between the number of dysregulated proteins and the relative decrease in SMN levels in SMA animals (Fig. 4E and Supplementary Figure S2). Differences in SMN levels between the different organs, control or SMA, are therefore unlikely to fully explain the organ-specific effects highlighted by the proteomics data, and rather suggest the presence of tissue/organ-specific pathways that are regulated by SMN during development.

## Discussion

In summary, we have generated novel, high-resolution proteomics datasets from SMA mouse embryos, facilitating detailed assessment of developmental aspects of the disease and SMN biology. Besides revealing the tissue-specific nature of SMN depletion during development, these datasets provide further support for a role for cytoskeletal dysregulation and the RhoA/ROCK signalling pathway in SMA (33,36,37). Of note, a recent paper by Siranosian *et al.* described changes in the actin cytoskeleton regulation pathway in whole blood transcriptome of adult SMA patients, confirming the systemic nature of pathway dysregulation in SMA (38). Combining μCT with these results, we present evidence suggesting that SMN depletion leads to a range of morphological and molecular developmental perturbations in SMA, manifesting in advance of the onset of degenerative neuromuscular symptoms commonly associated with the disease. It has long been known that SMN levels are higher prenatally than postnatally (8,39). Our findings therefore provide experimental support for the hypothesis that high levels of SMN during development highlight a strong requirement for the protein during prenatal stages of development and may help to explain, at least in part, some of the systemic alterations observed in SMA pathology (22). They also serve to reinforce the need for detailed studies of systemic pathologies in human patients, including those that are surviving for longer periods due to the action of new SMN-restoring therapies (5). Indeed, the magnitude of changes observed in the liver during the present study highlights the potential importance of this organ for understanding disease mechanisms and modulation (40–42).

Considering the number of dysregulated proteins identified in the liver, the finding that its volume was not significantly affected in the E14.5 Taiwanese embryos may be, initially, surprising. However, these results demonstrate that it is not possible to predict the presence or absence of molecular perturbations based solely on a morphological analysis of a tissue or organ, and vice versa. Our findings therefore highlight the importance of combining morphological and molecular assays to reveal the true extent of developmental phenotypes in SMA.

Our results may have significant implications for preclinical and clinical development and evaluation of therapies for SMA. Our finding of early changes in the SMA embryos of the Taiwanese mouse model provides further evidence for the presence and importance of an early therapeutic time window. Although these findings require validation in other mouse models of SMA, they extend our understanding of presymptomatic SMA. Our results suggest that effective delivery of therapy will likely require treatment in the presymptomatic phases of the disease, during the perinatal, or perhaps even prenatal, period in human patients (5,39,43). However, crucial ethical issues surround the use of human in utero gene therapy as a treatment strategy. Nevertheless, the heterogeneity of responses to SMN depletion that we observed across different tissues and organs serves to highlight the importance of careful systemic treatment delivery, and the limitations that may be associated with focusing solely on the neuromuscular system.

## Materials and Methods

### Experimental aims and design

This study was designed to determine the prenatal manifestations of Spinal Muscular Atrophy in the Taiwanese mouse model. All data presented here were replicated in at least three mice, with all volumetric and morphometric data quantified blinded to genotypes. The proteomics screen was performed on pooled samples coming from five embryos.

### Animals

All animals were bred and handled following the UK Animals (Scientific Procedures) Act, 1986. Procedures were approved by the internal ethics committee at the University of Edinburgh and following UK Home Office regulations. The Taiwanese model of SMA was maintained as previously described and obtained from Jackson Laboratories (strain 005058) (44). In the text, we refer to *Smn^+/−^;SMN2*^*tg/*0^ embryos as controls, and *Smn^−/−^;SMN2*^*tg/*0^ as SMA embryos. The morning of vaginal plug discovery following timed mating was counted as E0.5. The pregnant dam was culled, and the embryos collected when they reached the age of E14.5.

### Anatomy

#### Micro-computed tomography

A single litter of embryos was dissected at E14.5. Eight whole embryos (four controls and four SMA) were fixed in 4% paraformaldehyde (PFA) for 3 days at 4°C and then sent to the Experimental and Preclinical Imaging Centre at Leeds University. There, the embryos were incubated in 3 mL of Lugol’s (Sigma Aldrich, US) staining solution on a roller at room temperature. The staining solution was changed after 24 h. After 5 days, the embryos were embedded in 1% agarose (Web Scientific) and scanned with SkyScan 1176 (Bruker, Belgium) with the following parameters: 9 μm pixel size, 50 kV, 500 μA, 0.5 mm aluminium filter, exposure 1010 ms, rotation step 0.2°, 4 averages, 180° scan. The data were reconstructed with NRecon (Bruker) software using the Feldkamp algorithm with the following parameters: smoothing: 4, ring artefact correction: 14, beam hardening correction: 40%.

#### Volume measurements

Using Horos software (version 3.3.5), regions of interest (ROIs) were manually segmented in each individual transverse section and volumes were subsequently computed from these ROIs. All segmentation was performed blinded to the genotype of the embryos. Movies were obtained using the Movie Export tool (Supplementary Material, Movies 2–5).

#### Morphometric measurements of cardiac ventricles

Image orientation was adjusted in 3D-view in Horos to make consistent measurements across embryos. The transverse section chosen for measurements corresponded to the plane crossing the apex of the heart in sagittal view and perpendicular to the vertebral column of the embryo. Each value for the width of the IVS or the thickness of the left ventricular wall is the average length measured across the structure at three different locations, dorsoventrally. All morphometric analyses were performed blinded to the genotype of the embryos.

#### Surface rendering

Surface rendered images of whole embryos and internal organs were obtained using Amira (Amira 2019.3, ThermoFisher Scientific, US). Rendering was performed on whole embryo files downsampled by a 1:3 factor. The animation module of Amira was used to illustrate the segmentation and surface rendering processes (Supplementary Material, Movie 1).

### Histology

#### Cryotomy

Prior to sectioning, whole embryos from a single litter were dissected and fixed in 4% PFA overnight at 4°C. They were then left in a PBS-30% sucrose solution overnight and embedded in cryomolds using a 1:1 30% sucrose-OCT compound solution (embedding medium, VWR, US). Using a cryostat, all 20 μm thick transverse sections of the whole embryos were collected on Superfrost™Plus slides (ThermoFisher) and kept at −20°C prior to routine haematoxylin and eosin staining.

#### Morphometric measurements

IVS and left ventricular wall thickness were measured on the transverse sections where branching of the trachea into bronchi could be observed. Each value corresponds to the average length measured across the structure in three locations, dorsoventrally, from one embryo. Measurements were taken using Fiji (ImageJ, version 2.0.0). All morphometric analyses were performed blinded to the genotype of the embryos.

### Proteomics

E14.5 embryos from three different litters were individually dissected to collect spinal cord (ventral part) and brain cleaned of all meninges, as well as liver, heart and a sample of abdominal skeletal muscle. All samples were snap frozen on dry ice and then stored at −80°C prior to protein extraction. We used five SMA and five control samples pooled together for each organ, coming from these three litters.

#### Protein extraction

Individual organs were resuspended in label-free lysis buffer (100 nM Tris–HCl pH = 7.6, 4% (w/v) SDS) supplemented with 1 × EDTA-free protease inhibitor cocktail (ThermoFisher Scientific). The tissues were homogenized using GentleMACS™ M-Tubes on the protein run of the GentleMACS™ tissue dissociator (Miltenyi Biotech Inc., Germany). After transfer to Eppendorfs and centrifugation at 200 000 g for 20 min, the protein-containing supernatant was transferred to low-binding tubes and stored at −80°C.

#### Quality controls

All individual samples were quantified for protein concentration using a BCA assay and following manufacturer’s instructions (Pierce™ BCA Protein Assay Kit, ThermoFisher Scientific). To check for accurate quantification and sample integrity, for every sample, 5 μg of protein mixed with NuPAGE™ SDS sample buffer (4×, ThermoFisher) was loaded into the wells of an NuPAGE 4–12% Tris-Bis gel. After electrophoresis, the gels stained overnight at room temperature with InstantBlue™ total protein stain. The gels were then imaged using an infrared scanner (Odyssey LI-COR biosciences, US) at 700 nm and analyzed using ImageStudio™ Lite (LI-COR). After subtraction of background signal, fluorescence intensity was measured for each sample on the totality of the lane and on three arbitrarily chosen regions (198–62, 62–28 and 28–6 kDa) (45).

#### Pooling

For each organ, two pools were made, control and SMA, with each pool comprising of 25 μg of protein from five individual embryos. Quality controls were then performed on each of the 10 pools using BCA assays and total protein stains as described above (3 μg per pool were used for the electrophoresis.)

#### Mass spectrometry

The samples were processed for 10-plex TMT mass spectrometry at the FingerPrints Proteomics facility in Dundee, as previously described (46,47).

#### Data analysis

Filtered protein IDs were inputted into the IPA application (Ingenuity Systems, Silicon Valley, CA), which returned a set of mapped IDs, which were further sorted into significantly affected proteins based on a threshold of 20% of up- or downregulation (referred to as dysregulated proteins). A core analysis performed by IPA associates protein IDs in the Ingenuity’s Knowledge Database to identify affected canonical pathways. IPA returns a ratio of the number of molecules in the dataset per pathway and the total number of molecules that map to that pathway. To rank the top 100 most affected canonical pathways for each organ, we used the second value returned by IPA: the *P*-value of Fisher’s exact test (shown as -log(*P*-value)). Further computational analyses were performed in RStudio (version 1.2.1335) and GraphPad Prism (version 8.2.0). For protein overlap in absolute and percentage numbers, overlapping lists of proteins across two, three and four organs were extracted from Venny 2.1.0 (http://bioinfogp.cnb.csic.es/tools/venny/) and from IPA for all five organs. Bar charts were plotted in GraphPad and assembled in Affinity Designer (version 1.7.3) using the UpSet’s layout for visualization of intersections (48). Heatmaps for canonical pathways were built using the gplots library in RStudio. More information on the methodology behind IPA can be found at http://ingenuity.com/.

Further functional analyses on the differentially expressed proteins were performed using the DAVID, version 6.8, available at https://david.ncifcrf.gov/home.jsp. DAVID uses Fisher’s exact test to calculate *P*-values for protein enrichment scores. Enrichment for proteins in described categories was considered significant for Benjamini-corrected *P*-values <0.05 (49,50).

### Western blot

The samples used for proteomics were pooled again for quantitative western blotting. The same quantity of protein was used from each individually extracted sample. Pools were prepared for western blotting by adding 4 × SDS sample buffer containing β-mercaptoethanol (Sigma-Aldrich). For all pools, 10 μg of protein was loaded onto a 4–12% Bis-Tris gradient gel (ThermoFisher Scientific). The rest of the procedure followed what has been previously described (33,34). After transfer, membranes were incubated in Revert Total Protein Stain (LI-COR) for 1 h at room temperature, washed 2 × 5 min in Revert Total Protein wash solution (LI-COR) and 1 × 5 min in MilliQ water before blocking with Odyssey PBS blocking buffer (LI-COR) for 30 min at room temperature. Membranes were incubated overnight with 1:500 mouse anti-SMN antibody (BD bioscience 610 646) in blocking buffer. Membranes were then washed 3 × 10 min in PBS prior to incubation for 1 h at room temperature with a 1:5000 secondary antibody (donkey-anti-mouse IRDye 800, LI-COR) in blocking buffer. Membranes were washed 3 × 30 min in PBS, dried and imaged on an Odyssey CLX laser-based scanning system (LI-COR). Quantification was performed according to Groen et al. (32), by normalizing the SMN signal with the total protein stain signal of each lane.

### Statistics

Statistical analyses were performed in GraphPad Prism (version 8.2.0) using unpaired t-tests (significance ^*^P < 0.05, ^*^^*^P < 0.01) for volumetric and morphometric measurements.

### Data availability

The raw proteomics data supporting the conclusions of this article are available in the University of Edinburgh DataShare repository, https://doi.org/10.7488/ds/2776.

## Supplementary Material

Motyl_Et_Al_Supplementary_Material_FINAL_ddaa146

supp_movie1_surface_rendering_ddaa146

supp_movie2_whole_embryo_ddaa146

supp_movie3_brain_ddaa146

supp_movie4_liver_ddaa146

supp_movie5_cardiac_ventricles_ddaa146
