# Supplementary material for: Pre-natal manifestation of systemic developmental abnormalities in spinal muscular atrophy
Source: Hum Mol Genet. 2020 Jul 9;29(16):2674–83. doi: 10.1093/hmg/ddaa146 (PMC7530529; doi:10.1093/hmg/ddaa146)
Supplement: Motyl_Et_Al_Supplementary_Material_FINAL_ddaa146 [file motyl_et_al_supplementary_material_final_ddaa146.docx]

**Supplementary Material**

Supplementary Table 1. Summary of proteomics screen

| Mapped IDs: 6701 | |  |  |
| --- | --- | --- | --- |
| Organ | Dysregulated | Upregulated | Downregulated |
| Brain | 137 | 36 | 101 |
| Spinal cord | 194 | 35 | 159 |
| Liver | 1753 | 1 | 1752 |
| Heart | 193 | 34 | 159 |
| Muscle | 309 | 34 | 275 |

Supplementary Table 2. Proteins significantly regulated across all organs of interest

|  | GEMIN8 | DDX20/GEMIN3 | HPS4 |
| --- | --- | --- | --- |
| Brain | -2.075 | -1.263 | -2.436 |
| Spinal cord | -2.125 | -1.325 | -2.238 |
| Liver | -2.001 | -1.302 | -1.568 |
| Heart | -1.914 | -1.277 | 1.256 |
| Muscle | -2.192 | -1.373 | 1.325 |

Supplementary Table 3. Top 10 affected canonical pathways for each organ of interest returned by IPA

|  | **Canonical pathway** | **-log(p-value)** |
| --- | --- | --- |
| **Brain** | Hereditary Breast Cancer Signaling | 1.84 |
|  | Retinoate Biosynthesis II | 1.62 |
|  | Tetrapyrrole Biosynthesis II | 1.53 |
|  | Telomerase Signaling | 1.43 |
|  | NAD Biosynthesis from 2-amino-3-carboxymuconate Semialdehyde | 1.38 |
|  | Histidine Degradation III | 1.33 |
|  | Heme Biosynthesis II | 1.28 |
|  | PXR/RXR Activation | 1.23 |
|  | Calcium Transport I | 1.23 |
|  | IL-12 Signaling and Production in Macrophages | 1.22 |
| **Spinal cord** | Leukocyte Extravasation Signaling | 3.23 |
|  | PFKFB4 Signaling Pathway | 3.16 |
|  | B Cell Receptor Signaling | 2.75 |
|  | Glucose and Glucose-1-phosphate Degradation | 2.4 |
|  | Chemokine Signaling | 2.27 |
|  | NGF Signaling | 2.27 |
|  | Synaptic Long Term Potentiation | 2.26 |
|  | Role of IL-17F in Allergic Inflammatory Airway Diseases | 2.15 |
|  | PI3K Signaling in B Lymphocytes | 2.13 |
|  | Regulation of IL-2 Expression in Activated and Anergic T Lymphocytes | 2.11 |
| **Liver** | Axonal Guidance Signaling | 15.7 |
|  | Synaptogenesis Signaling Pathway | 11.4 |
|  | Ephrin Receptor Signaling | 10.2 |
|  | Protein Kinase A Signaling | 8.84 |
|  | Thrombin Signaling | 8.66 |
|  | 14-3-3-mediated Signaling | 7.53 |
|  | Actin Cytoskeleton Signaling | 7.51 |
|  | Signaling by Rho Family GTPases | 7.48 |
|  | Apelin Cardiomyocyte Signaling Pathway | 7.43 |
|  | CXCR4 Signaling | 7.26 |
| **Heart** | GABA Receptor Signaling | 2.04 |
|  | Methionine Degradation I (to Homocysteine) | 1.82 |
|  | Cysteine Biosynthesis III (mammalia) | 1.75 |
|  | Gluconeogenesis I | 1.68 |
|  | 4-aminobutyrate Degradation I | 1.59 |
|  | Cellular Effects of Sildenafil (Viagra) | 1.57 |
|  | Uracil Degradation II (Reductive) | 1.47 |
|  | Thymine Degradation | 1.47 |
|  | Aryl Hydrocarbon Receptor Signaling | 1.45 |
|  | Superpathway of Methionine Degradation | 1.4 |
| **Muscle** | FXR/RXR Activation | 15.2 |
|  | LXR/RXR Activation | 13.2 |
|  | Acute Phase Response Signaling | 9.35 |
|  | GP6 Signaling Pathway | 8.29 |
|  | Hepatic Fibrosis / Hepatic Stellate Cell Activation | 8.2 |
|  | Coagulation System | 7.72 |
|  | Atherosclerosis Signaling | 6.71 |
|  | Calcium Signaling | 5.99 |
|  | Cellular Effects of Sildenafil (Viagra) | 5.72 |
|  | Epithelial Adherens Junction Signaling | 5.06 |


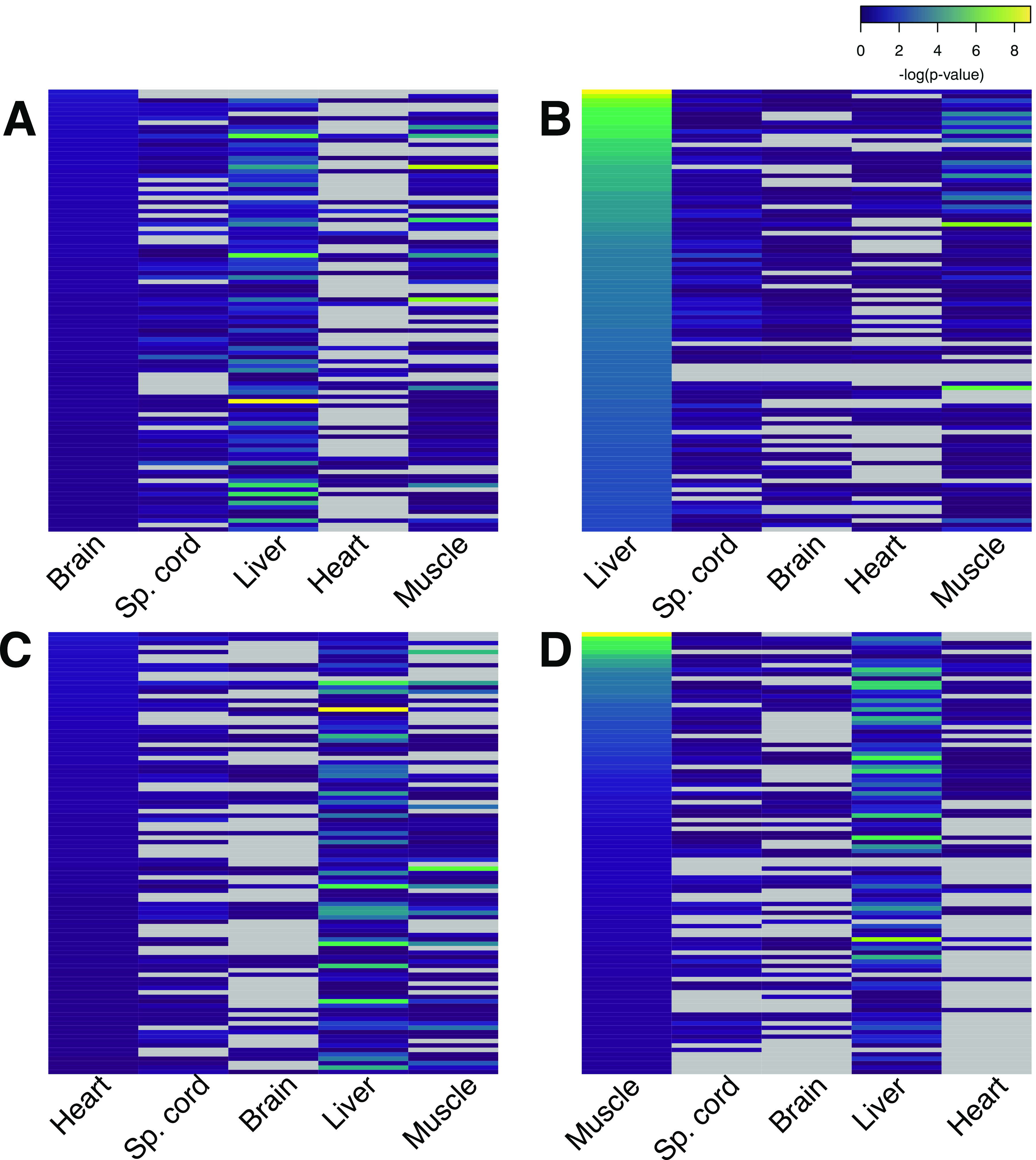


**Supplementary Figure 1. Organ/tissue-specific molecular pathway disruption.** Heatmaps anchored on the top 100 affected canonical pathways of the Brain (A), Liver (B), Heart (C) and Muscle (D), according to -log(p-value) (Fisher’s exact test) for each pathway identified, as generated by IPA. Pathways for which p-values could not be calculated in given organs are shown in grey. Note the lack of consistency of changes across each of the different tissues/organs.


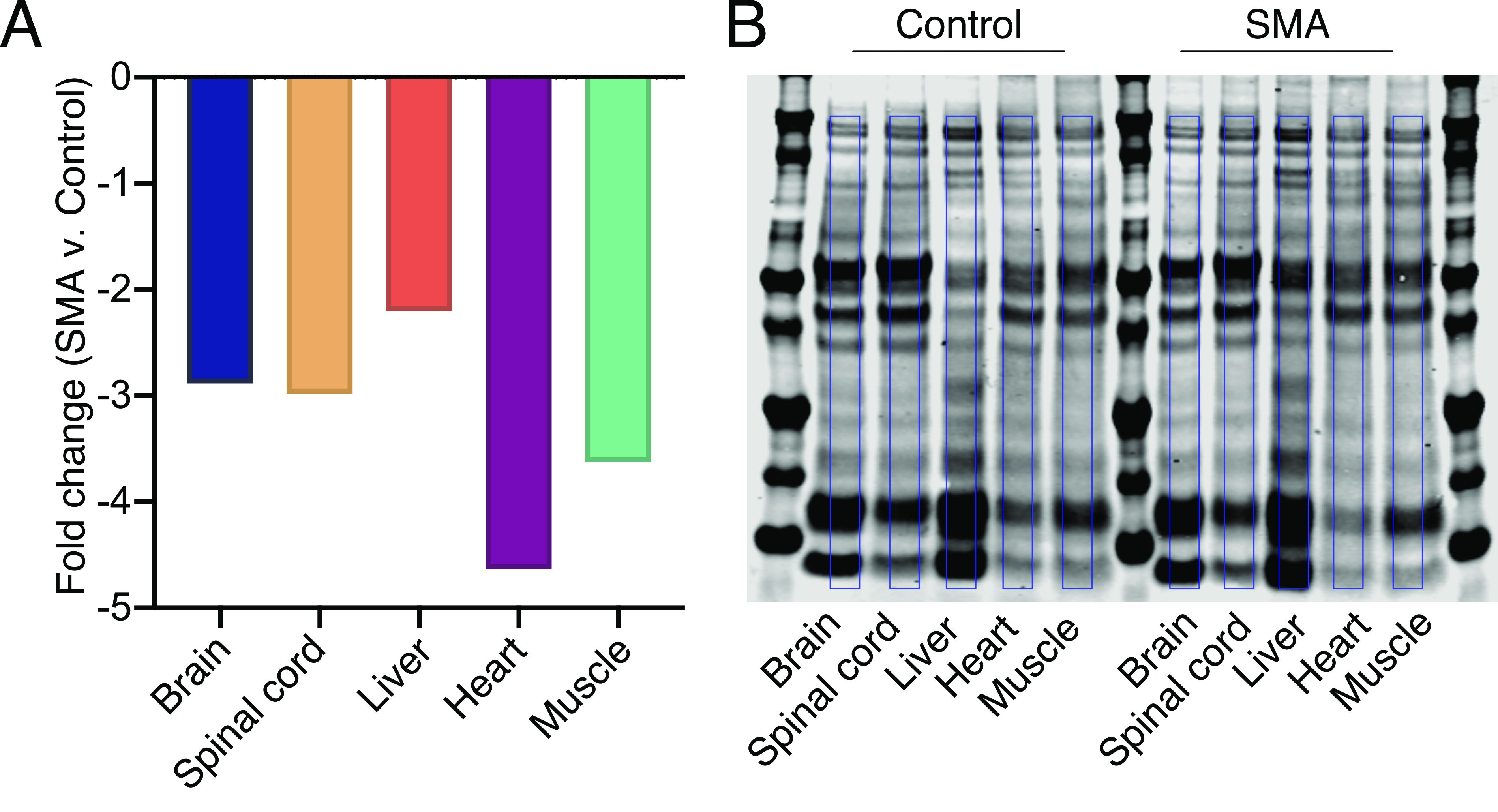


**Supplementary Figure 2. Organ/Tissue-specific expression of SMN.** (A) Bar chart showing the fold changes of SMN protein levels in SMA v. Control embryos as measured by quantitative western blot. (B) Fluorescent Total Protein Stain (TPS) used for SMN protein quantification.

Supplementary movie legends

Supplementary movie 1. E14.5 Control v. SMA embryos µCT surface-rendering, scale bars = 2 mm

Supplementary movie 2. E14.5 Control v. SMA, whole embryo segmentation

Supplementary movie 3. E14.5 Control v. SMA, brain segmentation

Supplementary movie 4. E14.5 Control v. SMA, liver segmentation

Supplementary movie 5. E14.5 Control v. SMA, cardiac ventricle segmentation
